# Effect of Pokémon Go on Self-Harm Using Population-Based Interrupted Time-Series Analysis: Quasi-Experimental Study

**DOI:** 10.2196/17112

**Published:** 2020-06-12

**Authors:** Rosa Sze Man Wong, Frederick Ka Wing Ho, Keith Tsz Suen Tung, King-Wa Fu, Patrick Ip

**Affiliations:** 1 Department of Paediatrics and Adolescent Medicine University of Hong Kong Hong Kong China (Hong Kong); 2 Institute of Health and Wellbeing University of Glasgow Glasgow United Kingdom; 3 Journalism and Media Studies Centre University of Hong Kong Hong Kong China (Hong Kong)

**Keywords:** Pokémon Go, self-harm, mobile game, injury, mHealth interventions, interrupted time-series analysis

## Abstract

**Background:**

Pokémon Go is a very popular location-based augmented reality game with widespread influences over the world. An emerging body of research demonstrates that playing Pokémon Go can lead to improvements in physical activity and psychosocial well-being; however, whether Pokémon Go reduces self-harm incidence at the population-level is still questionable.

**Objective:**

This study aimed to quantify how the launch of Pokémon Go in Hong Kong affected the incidence of self-harm using a quasi-experimental design.

**Methods:**

An interrupted time-series design with Poisson segmented regression adjusted for time and seasonality trends was used on data from 2012 to 2018 to detect any changes in the number of accident and emergency attendances due to self-harm, after Pokémon Go was launched. The findings were validated using a baseline control period and using other intentional injuries and minor noninjuries as control outcomes. We also assessed intervention effects by age group.

**Results:**

From January 1, 2012 to July 31, 2018, there were 13,463 accident and emergency attendances due to self-harm in Hong Kong. During the period after launching Pokémon Go, self-harm attendances dropped by 34% (adjusted incidence rate ratio: 0.66, 95% CI 0.61-0.73). When analyzed by age group, a reduction in self-harm incidence was only apparent in adults (18 to 24 years of age: adjusted incidence rate ratio: 0.78, *P*=.02; 25 to 39 years of age: adjusted incidence rate ratio: 0.75, *P*<.001; 40 years of age and older: adjusted incidence rate ratio: 0.57, *P*<.001).

**Conclusions:**

Self-harm incidence in the population, particularly in adults, showed a significant decline in the period after Pokémon Go was launched. Augmented reality games such as Pokémon Go show great promise as a tool to enhance psychosocial well-being and improve mental health.

## Introduction

Self-harm refers to an act of causing physical injury to oneself without suicidal intent (for example, self-cutting, scratching oneself, hitting oneself, or intentional drug overdose). Cumulative evidence supports that a significant proportion of adolescents and adults have intentionally harmed themselves during their lifetime. Annually, in the United Kingdom, general hospitals have more than 200,000 cases related to individuals who self-harm [1]. In Hong Kong, research has found an alarming prevalence of self-harm in the male (13.4%) and female adolescent (19.7%) populations [2]. In some countries, the incidence of self-harm continues to rise steadily [3,4]. Although estimates of incidence vary among countries [5-8], self-harm is a global public health concern because of its association with suicide [9-11]. Repeated self-harm is common, with 16% and 23% repeating self-harm within one year and four years, respectively [12]. Given the substantial economic and health consequences, effective preventive interventions are urgently needed to decrease the incidence of self-harm. Research has shown that self-harm may signal distress or may be used as a way to escape from a troubling situation [6,13]. Psychosocial characteristics such as elevated stress, depression, anxiety, and feelings of lack of belonging, social isolation, or low self-esteem are common in people who self-harm [14,15]. Interventions that attempt to reduce distress and improve interpersonal skills offer promise in reducing self-harm frequency and repeated self-harm incidents [16]. Individual or group-based psychotherapy (problem-solving training, skills training, and cognitive modification training) and family therapy are typical psychosocial interventions to address self-harm [17].

Unfortunately, most people who harm themselves are reluctant either to admit that they self-harm or to seek help prior to harming themselves [18]. Hence, traditional face-to-face interventions may help only a portion of those at-risk (such as individuals who are more accepting of their mental health problems). In recent years, public health projects have used mobile technology to improve health and well-being [19-21]. Such technological advancements have extended the scope of service delivery to diverse and hidden populations. There has been a proliferation of novel interventions that incorporate technological innovation to empower young people to cope with difficult situations [22-24]. The rise of augmented reality games can improve player experience through the integration of game elements with real-world elements [25]. Notably, Pokémon Go, a location-based augmented reality game, has been widely successful around the world. The game uses geolocation to motivate players to search in real-world locations to capture virtual creatures called Pokémon. Since Pokémon appear at a location in a random sequence and for a limited period of time, players are required to visit different places to catch them to progress in the game.

Pokémon Go has attracted millions of players; in 2018, around 150 million people were actively playing the game. It has also prompted research into and discussion of the benefits and drawbacks of the game. Previous studies [26,27] have reported that the use of Pokémon Go has led to increases in physical activity. It has been found to promote social interaction, but also to increase the risk of injury and violence [28]. In Japan, the incidence of fatal traffic injuries did not change significantly after the release of Pokémon Go [29]. A few anecdotal reports and some evidence have investigated the positive effects of Pokémon Go on psychosocial well-being such as improving mood, expanding social network, and prompting individuals who mostly remain indoors to venture outside [30,31]. Given that the etiology of self-harm is complex and multifactorial involving numerous factors in physical, social, and mental health domains [32], Pokémon Go may also benefit multiple areas. The game has great potential to reduce the risk of self-harm; however, the effectiveness of Pokémon Go in preventing self-harm has not been empirically determined.

In Hong Kong, Pokémon Go was released on July 25, 2016. After the game was launched, many people in Hong Kong were drawn outdoors and to public places to play. In contrast to places of low population density, players in densely populated areas such as Hong Kong are more likely to interact with other players while playing Pokémon Go. The opportunity to engage casually with others in public has been found to mitigate feelings of loneliness because of an increased sense of belonging in the community [33]. Previous studies [30,34,35] focus on individual-level changes in physical activity, and little is known about the influences of Pokémon Go at the population level especially in Hong Kong where population is large but public spaces are limited; therefore, we carried out a quasi-experimental evaluation to examine any changes in the incidences of self-harm and other injury and noninjury events in Hong Kong after the launch of Pokémon Go.

## Methods

### Data Source and Participants

We extracted patient data from the Accident and Emergency Information System of the Hong Kong Hospital Authority. The Hong Kong Hospital Authority is the official managing body of public hospitals in Hong Kong and is responsible for most 24-hour accident and emergency services within the territory. In this study, data representing patients aged 12 years and older who presented to the accident and emergency department with evidence of intentional or minor noninjury events from January 1, 2012 to July 31, 2018 and from January 1, 2002 to July 31, 2008 were included. Codes from the International Classification of Diseases (ninth revision) were used to retrieve accident and emergency attendance records. The primary outcome was accident and emergency attendance for self-harm (codes E950 through E959). To validate the effect of Pokémon Go on self-harm incidence, we used two control outcomes [36]: injury events inflicted by others (common assault, indecent assault, and abuse or battering; codes E960 through E969) and minor noninjury events (all cases in nontrauma triage category 5) prior to and after the game’s launch. Data were extracted from General Household Surveys and Population Census/By-census from the Hong Kong Census and Statistics Department.

### Statistical Analysis

We used interrupted time-series analysis with a slope change model (equation 1) to detect changes in accident and emergency outcomes after the launch of Pokémon Go. A slope change model was selected because injury outcomes such as self-harm involve a complex causal mechanism that could result in gradual change over time [37]. Interrupted time series analysis is a quasi-experimental method used frequently in natural experiments to investigate change in the level of the outcome before and after an intervention. In this study, we defined the primary baseline control period as January 1, 2012 to July 24, 2016 (the day before Pokémon Go was released) and the primary exposure period as July 25, 2016 (the day that Pokémon Go was released) to July 31, 2018. The negative control exposure period was defined as July 25, 2006 to July 31, 2008 and the negative control baseline period was defined as January 1, 2002 to July 24, 2006. The incidence rate ratio was calculated using Poisson regression with robust standard errors with adjustments for age, sex, seasonality, and time trends. The incidence rate in the baseline control period was set as the reference value. Repeated accident and emergency attendances were removed prior to analysis. The model used accident and emergency attendance counts as the dependent variable and the time after intervention period as the primary independent variable. Population size was controlled as an offset variable to convert the monthly accident and emergency attendance counts into a rate value to control for change in the population over time [38]. The model is described by:

ln(*y*_t_) = ln(*population*_t_)+ *β*_0_ + *β*_1_ * *time*_t_ + *β*_2_ * *time after intervention*_t_ + *β*_3_(age group) + *β*_4_(sex) + *β*_5_(seasonality)+ *e*_t_ (1)

where *time*_t_ is the number of months from the start of the observation period, *time after intervention*_t_ is the number of months after the intervention, *y*_t_ is the outcome at *time*_t_, ln(*population*_t_) is an offset used to account for the population at *time*_t_, *β*_0_ is a coefficient that estimates the baseline level of the outcome; *β*_1_ is a coefficient that estimates trends over time; *β*_2_ is a coefficient that estimates the change in the trend after the intervention ; *β*_3_ is a coefficient that accounts for age, *β*_4_ is a coefficient that accounts for sex, *β*_5_ is a coefficient that accounts for seasonality, and *e*_t_ is the random effect at *time*_t_. The coefficient of the slope term, *β*_2_, represents the incidence rate ratio, which is a measure of relative risk. An incidence rate ratio of 1 indicated the association between the intervention (the release of Pokémon Go) and the change in accident and emergency attendance was null. An incidence rate ratio greater than 1 indicated a higher risk of self-harm in the period, whereas an incidence rate ratio less than 1 indicates a lower risk of self-harm in the period. In addition, we also compared the change in slope by age group and between early (from July 25, 2016 to July 24, 2017) and late exposure (July 25, 2017 to July 31, 2018) periods. All analyses were conducted using R statistical software (version 3.5.2), with *P*<.05 indicating statistical significance.

## Results

Table 1 displays summary statistics for the entire study period. Of 13,463 accident and emergency attendances attributed to self-harm recorded for the period from January 1, 2012 to July 31, 2018, 7521 (55.9%) were female, 5942 (44.1%) were male, and most episodes (6898, 51.2%) occurred in patients aged 40 years or older. In the same period, there were 85,957 and 1,972,805 other intentional injuries and minor noninjuries, respectively.

**Table 1:** Accident and emergency attendance in the observation period (from January 1, 2012 to July 31, 2018).

Accident and emergency attendance		Self-harm, n (%)	Other intentional injury^a^, n (%)	Minor noninjury^b^, n (%)
Total		13,463 (100)	85,957 (100)	1,972,805 (100)
**Gender**				
	Female	7521 (55.9)	32,378 (37.7)	912,587 (46.3)
	Male	5942 (44.1)	53,579 (62.3)	1,060,218 (53.7)
**Age (years)**				
	12-17	1109 (8.2)	5858 (6.8)	99,064 (5.0)
	18-24	1822 (13.5)	9636 (11.2)	168,629 (8.5)
	25-39	3634 (27.0)	27,740 (32.3)	431,028 (21.8)
	≥40	6898 (51.2)	42,723 (49.7)	1,274,084 (64.6)

^a^Common assault, indecent assault, and abuse or battering.

^b^Minor noninjury accident and emergency attendance.

Table 2 shows that in the period following the launch of Pokémon Go, self-harm attendances decreased by 34% (incidence rate ratio 0.66, *P*<.001). In contrast, accident and emergency attendances related to other intentional injuries and minor noninjuries did not show declining trends after the game was launched. Furthermore, during the negative control exposure period in which Pokémon Go was absent, an increasing trend of self-harm attendances was found.

**Figure 1:**
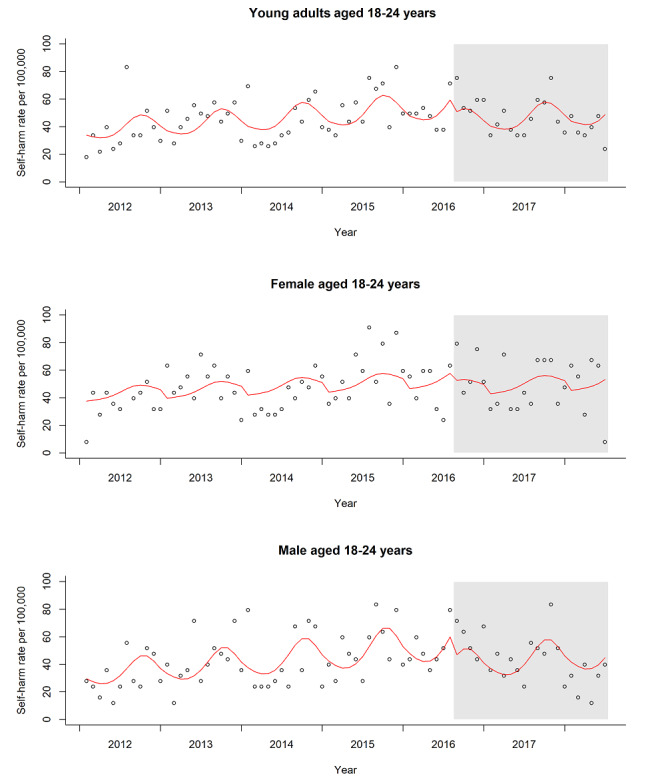
Monthly self-harm incidence rate of young adults in the observation period.

Attendances that were related to common assault demonstrated an increase during both the postlaunch and baseline periods. In age-specific analyses, from the immediate postlaunch to the end of the observation period, decreasing trends of self-harm attendances were only detected in adults (18 years and older). Specifically, the trend in self-harm attendances showed declines of 22% (incidence rate ratio 0.78, 95% CI 0.63-0.95) for ages 18 to 24 years, 25% (incidence rate ratio 0.75, 95% CI 0.66-0.87) for ages 25 to 39 years, and 43% (incidence rate ratio 0.57, 95% CI 0.49-0.67) for 40 years of age and older. Figure 1 illustrates self-harm incidence rate trends for ages 18 to 24 years in the observation period.

**Table 2:** Association between Pokémon Go and accident and emergency attendance from robust Poisson regression with adjustments for time trends, seasonal trends, age, gender, and population size.

Outcomes			Primary exposure period^a^		Negative control exposure period^b^		
			IRR^c^ (95% CI)	*P* value		IRR (95% CI)	*P* value
**All**							
	**Primary outcome**						
		Self-harm	0.66 (0.61, 0.73)	<.001		1.74 (1.57, 1.92)	<.001
	**Negative control outcomes**						
		Common assault	1.06 (1.01, 1.11)	.03		1.05 (1.00, 1.10)	.04
		Indecent assault	1.01 (0.81, 1.26)	.93		1.11 (0.92, 1.35)	.28
		Abuse/battering	1.11 (1.00, 1.24)	.06		1.04 (0.94, 1.16)	.41
		Minor noninjury	1.11 (1.03, 1.19)	.007		—	—
**12-17 years of age**							
	**Primary outcome**						
		Self-harm	0.82 (0.65, 1.04)	.10		1.39 (1.11, 1.73)	.004
	**Negative control outcomes**						
		Common assault	1.21 (1.03, 1.42)	.02		1.13 (1.02, 1.25)	.02
		Indecent assault	0.97 (0.54, 1.77)	.93		1.34 (0.82, 2.20)	.24
		Abuse/battering	1.14 (0.80, 1.64)	.47		1.08 (0.83, 1.40)	.57
		Minor noninjury	1.17 (1.07, 1.28)	<.001		1.13 (1.02, 1.25)	.02
**18-24 years of age**							
	**Primary outcome**						
		Self-harm	0.78 (0.63, 0.95)	.02		1.32 (1.08, 1.60)	.006
	**Negative control outcomes**						
		Common assault	0.99 (0.90, 1.09)	.90		1.03 (0.96, 1.10)	.47
		Indecent assault	1.34 (0.86, 2.10)	.20		0.96 (0.64, 1.43)	.84
		Abuse/battering	1.35 (0.88, 2.08)	.17		1.32 (0.92, 1.90)	.13
		Minor noninjury	1.12 (1.07, 1.18)	<.001		1.32 (1.08, 1.60)	.006
**25-39 years of age**							
	**Primary outcome**						
		Self-harm	0.75 (0.66, 0.87)	<.001		1.64 (1.39, 1.92)	<.001
	**Negative control outcomes**						
		Common assault	1.02 (0.96, 1.08)	.51		1.03 (0.98, 1.08)	.26
		Indecent assault	1.15 (0.81, 1.64)	.44		1.12 (0.78, 1.61)	.55
		Abuse/battering	1.07 (0.91, 1.25)	.41		1.01 (0.89, 1.14)	.89
		Minor noninjury	1.07 (1.02, 1.12)	.004		1.03 (0.98, 1.08)	.26
**≥40 years of age**							
	**Primary outcome**						
		Self-harm	0.57 (0.49, 0.67)	<.001		2.15 (1.76, 2.62)	<.001
	**Negative control outcomes**						
		Common assault	1.07 (1.01, 1.12)	.01		1.05 (1.00, 1.10)	.046
		Indecent assault	0.79 (0.55, 1.14)	.20		1.11 (0.81, 1.53)	.51
		Abuse/battering	1.12 (0.99, 1.27)	.08		1.05 (0.93, 1.18)	.47
		Minor noninjury	1.11 (1.05, 1.16)	<.001		2.15 (1.76, 2.62)	<.001

^a^Primary exposure period: July 25, 2016 to July 31, 2018; control baseline period: January 1, 2012 to July 24, 2016.

^b^Negative control exposure period: July 25, 2006 to July 31, 2008; negative control baseline period: January 1, 2002 to July 24, 2006.

^c^IRR: incidence rate ratio.

Table 3 shows the immediate and delayed effects of Pokémon Go launch on the trends of accident and emergency attendances. Both early and late exposure periods had negative slopes representing decreasing self-harm incidence, but the decrease was greater in the late exposure period than in the early exposure period overall (late: incidence rate ratio 0.57, *P*<.001; early: incidence rate ratio 0.71, *P*<.001) and for the adult age groups (late: incidence rate ratio 0.45-0.70, *P*<.001 to *P*=.007; early: incidence rate ratio 0.63-0.80, *P*<.001 to *P*=.05).

**Table 3:** Association between accident and emergency attendance and early and late periods after the launch of Pokémon Go from robust Poisson regression with adjustments for time trends, seasonal trends, age, gender, and population size.

Outcomes			Early exposure period^a^		Late exposure period^b^		
			IRR^c^ (95% CI)	*P* value		IRR (95% CI)	*P* value
**All**							
	**Primary outcome**						
		Self-harm	0.71 (0.65, 0.78)	<.001		0.57 (0.51, 0.63)	<.001
	**Negative control outcomes**						
		Common assault	1.05 (0.99, 1.10)	.08		1.08 (1.02, 1.14)	.01
		Indecent assault	1.02 (0.81, 1.29)	.85		0.98 (0.75, 1.29)	.90
		Abuse/battering	1.08 (0.97, 1.22)	.17		1.17 (1.02, 1.34)	.02
		Minor noninjury	1.09 (1.01, 1.18)	.02		1.14 (1.04, 1.25)	.005
**12-17 years of age**							
	**Primary outcome**						
		Self-harm	0.78 (0.60, 1.00)	.05		0.92 (0.69, 1.22)	.56
	**Negative control outcomes**						
		Common assault	1.09 (0.92, 1.29)	.30		1.47 (1.22, 1.78)	<.001
		Indecent assault	0.92 (0.48, 1.76)	.79		1.10 (0.52, 2.31)	.81
		Abuse/battering	0.98 (0.66, 1.47)	.93		1.51 (0.98, 2.34)	.07
		Minor noninjury	1.15 (1.04, 1.26)	.005		1.22 (1.09, 1.36)	<.001
**18-24 years of age**							
	**Primary outcome**						
		Self-harm	0.81 (0.66, 1.00)	.05		0.70 (0.55, 0.91)	.007
	**Negative control outcomes**						
		Common assault	1.01 (0.91, 1.11)	.91		0.97 (0.86, 1.09)	.60
		Indecent assault	1.31 (0.82, 2.11)	.26		1.41 (0.81, 2.48)	.23
		Abuse/battering	1.35 (0.86, 2.12)	.19		1.35 (0.78, 2.33)	.29
		Minor noninjury	1.11 (1.05, 1.17)	<.001		1.16 (1.08, 1.23)	<.001
**25-39 years of age**							
	**Primary outcome**						
		Self-harm	0.80 (0.70, 0.92)	.003		0.66 (0.55, 0.78)	<.001
	**Negative control outcomes**						
		Common assault	1.02 (0.96, 1.09)	.46		1.01 (0.94, 1.09)	.76
		Indecent assault	1.09 (0.75, 1.59)	.65		1.31 (0.85, 2.02)	.22
		Abuse/battering	1.06 (0.90, 1.25)	.50		1.09 (0.89, 1.33)	.40
		Minor noninjury	1.06 (1.01, 1.12)	.01		1.09 (1.03, 1.16)	.005
≥**40 years of age**							
	**Primary outcome**						
		Self-harm	0.63 (0.54, 0.73)	<.001		0.45 (0.38, 0.54)	<.001
	**Negative control outcomes**						
		Common assault	1.05 (1.00, 1.11)	.05		1.09 (1.03, 1.16)	.005
		Indecent assault	0.87 (0.61, 1.26)	.47		0.60 (0.38, 0.95)	.03
		Abuse/battering	1.10 (0.96, 1.25)	.17		1.17 (1.01, 1.36)	.04
		Minor noninjury	1.09 (1.04, 1.15)	.001		1.15 (1.08, 1.22)	<.001

^a^Early exposure period: July 25, 2016 to July 24, 2017; control baseline period: January 1, 2012 to July 24, 2016.

^b^Late exposure period: July 25, 2017 to July 31, 2018; control baseline period: January 1, 2012 to July 24, 2016.

^c^IRR: incidence rate ratio.

## Discussion

### Principal Findings

Given the substantial health care costs of self-harm, it is imperative to address and attempt to alleviate psychosocial difficulties before individuals harm themselves. Although Pokémon Go has been studied in outdoor settings for physical activity outcomes [26,27,30,34,35], there has been limited research on the effects of Pokémon Go on mental health (in particular, on its potential as a tool for the prevention of mental health disorders). Pokémon Go may lead to decreases in the frequency of self-harm by encouraging behaviors that improve mental health; however, no prior research has tested this hypothesis. This study is the first to assess and compare self-harm incidence using population-level data from before and after the release of Pokémon Go.

Based on hospital accident and emergency attendance records, after the release of Pokémon Go, decreasing trends for self-harm attendance records were found overall and in adult age groups (18 to 24 years of age, 25 to 39 years of age , and 40 years of age and older). Furthermore, the decline was larger in older ages, possibly because, as it has been suggested in various reports [30,39,40], adolescents are less likely to engage in Pokémon Go, and thus, this age group may be less affected by the game. In addition to analyses by age, analyses by exposure period showed that the effect of Pokémon Go’s launch on trends of self-harm attendances was more apparent in the late exposure period. This could be because protective factors, such as enhanced positive emotions, new friendships, and increased physical activity, that improve mental health occur and accumulate over time. To confirm the effect of Pokémon Go with decreased self-harm attendances, we repeated the analyses using the number of accident and emergency attendances due to other intentional injury and noninjury events as control outcomes and using the number of accident and emergency attendances for the 10 years before the introduction of Pokémon Go as a control period. This period (starting 10 years before the primary exposure) was selected as the control period because during this period of time, the use of information technology in health research was still low [41]. We found that the declining trend of self-harm attendances occurred only in the postlaunch period, whereas other accident and emergency attendances showed no decreases in both primary exposure (postlaunch) and negative exposure periods. These findings support the claim that playing Pokémon Go is a potential preventive strategy for self-harm [30,31]. Among the outcomes, only self-harm attendances showed significant declines in the postlaunch period, possibly because gameplay may have contributed to increased optimism, excitement, and positive social interactions [42]. The finding that the trends of accident and emergency attendances due to other intentional injury and minor noninjury events did not decline during the same period supports this assumption.

Notably, the increasing trend of common assault attendances in the postlaunch period may support the claim that Pokémon Go may trigger violence [28]. Analyses by age showed an increasing trend for common assault attendances in the age groups of 12 to 17 years and 40 years and older, but the trends remained largely unchanged in young adults (ages 18 to 39 years). This finding might be explained, among other factors, by player perception. A study [43] found that older adults had more negative attitudes about playing Pokémon Go and were more likely to agree that the game encourages violence. Although more research is needed to elucidate mechanisms, biases and differences in these game-related perceptions may lead to increased hostile and aggressive behavior between players and nonplayers [44], thereby increasing the chance of assaults. Previous research [45] also found that people under 18 years of age, when playing Pokémon Go, had the greatest tendency to behave badly (for example, by violating rules and regulations, threatening the safety of others, or venturing into unsafe places) which may have given rise to the increased number of assaults in this age group. Although our findings shed some light on the effect of Pokémon Go on violent behavior, future investigations should ascertain if young adults are less susceptible to the negative influences from Pokémon Go. Research should also identify protective factors to disengage players from aggressive and illegal behavior during the gameplay.

### Comparison with Prior Work

Augmented reality technology has been increasingly adopted, with promising results, in health interventions to promote physical and mental health [46,47]; however, there is a lack of evidence that confirms the effect of game-based interventions on suicide prevention. Only a few studies have investigated the benefits of playing Pokémon Go on mental health and have reported that playing Pokémon Go, both alone and with others, can positively affect the players’ well-being and social life [48], possibly by helping players shut out negative thoughts or concerns through flow experience—a state of complete absorption or engagement in the game activity [49,50]. Research shows that flow experience is linked to positive affect [51], and increased levels of flow experience were found in both solo and collaborative gameplay [52,53]. Some studies [48,52] have found that playing alone was a stronger predictor of flow experience, whereas other studies [53,54] have suggested collaborative play may have greater potential to trigger positive emotional and nonviolent responses. Although this study found a significant decrease in self-harm attendances after the release of Pokémon Go, it is important to highlight that the evidence was based upon aggregate data. Aggregated data can reflect both individual-level gaming behavior and societal factors such as public events and overall atmosphere during the period of exposure (the period after the Pokémon Go game was launched). Furthermore, while video games have a potential to reduce social isolation and psychological distress [46,55,56], some studies [55] have reported that problematic use of video games can cause disorder-related symptoms. The question of how frequently and where one should play Pokémon Go to reduce self-harm is worthy of exploration in future research.

### Limitations

The main limitation of this study was its ecological design. As our analyses were based on aggregate data, we could not identify whether the people presenting to accident and emergency due to self-harm had played Pokémon Go, and hence, we could not generalize our findings from the population-level to the individual-level; however, our results were consistent with previous ecological and observational study findings. Because of Pokémon Go’s large number of downloads and wide popularity within the community, the game has become an ongoing social event that extends to every part of Hong Kong. Hong Kong is a densely populated city where social events or movements have pervasive and substantial effects on mental health across socioeconomic classes and which may include effects on individuals who did not directly participate [57]. It should be noted that our analyses did not take into account the change in social atmosphere due to other social events in Hong Kong occurring during the same period which may have biased our results. Nevertheless, when replicating the analyses using other intentional injury and minor noninjury outcomes and earlier exposure periods that were not be influenced by the release of Pokémon Go, we found no significant decline which supports the conclusion that the decline in self-harm rate after the launch of Pokémon Go was not as a result of unobserved confounding.

### Conclusions

Augmented reality games are designed to bring the fun and exciting elements of virtual and natural environments together. The gameplay may create new and improved perceptions of everyday activities in the real world, thereby replacing intrusive and repetitive self-harm thoughts with positive feelings in players. This study found significant declines in self-harm incidence after the release of Pokémon Go. Future research should explore these possibilities and confirm the underlying mechanisms. We see promise in utilizing augmented reality games such as Pokémon Go with engaging and appealing features to reach large numbers of people and to improve their mental health.
